# A systems biology approach reveals common metastatic pathways in osteosarcoma

**DOI:** 10.1186/1752-0509-6-50

**Published:** 2012-05-28

**Authors:** Ricardo J Flores, Yiting Li, Alexander Yu, Jianhe Shen, Pulivarthi H Rao, Serrine S Lau, Marina Vannucci, Ching C Lau, Tsz-Kwong Man

**Affiliations:** 1 Texas Children’s Cancer and Hematology Centers, Texas Children’s Hospital, Houston, TX, USA; 2 Department of Pediatrics, Baylor College of Medicine, Houston, TX, USA; 3 Dan L. Duncan Cancer Center, Baylor College of Medicine, Houston, TX, USA; 4 Southwest Environmental Health Science Centers, The University of Arizona, Tucson, AZ, USA; 5 Department of Statistics, Rice University, Houston, TX, USA

## Abstract

**Background:**

Osteosarcoma (OS) is the most common malignant bone tumor in children and adolescents. The survival rate of patients with metastatic disease remains very dismal. Nevertheless, metastasis is a complex process and a single-level analysis is not likely to identify its key biological determinants. In this study, we used a systems biology approach to identify common metastatic pathways that are jointly supported by both mRNA and protein expression data in two distinct human metastatic OS models.

**Results:**

mRNA expression microarray and N-linked glycoproteomic analyses were performed on two commonly used isogenic pairs of human metastatic OS cell lines, namely HOS/143B and SaOS-2/LM7. Pathway analysis of the differentially regulated genes and glycoproteins separately revealed pathways associated to metastasis including cell cycle regulation, immune response, and epithelial-to-mesenchymal-transition. However, no common significant pathway was found at both genomic and proteomic levels between the two metastatic models, suggesting a very different biological nature of the cell lines. To address this issue, we used a topological significance analysis based on a “shortest-path” algorithm to identify topological nodes, which uncovered additional biological information with respect to the genomic and glycoproteomic profiles but remained hidden from the direct analyses. Pathway analysis of the significant topological nodes revealed a striking concordance between the models and identified significant common pathways, including “Cytoskeleton remodeling/TGF/WNT”, “Cytoskeleton remodeling/Cytoskeleton remodeling”, and “Cell adhesion/Chemokines and adhesion”. Of these, the “Cytoskeleton remodeling/TGF/WNT” was the top ranked common pathway from the topological analysis of the genomic and proteomic profiles in the two metastatic models. The up-regulation of proteins in the “Cytoskeleton remodeling/TGF/WNT” pathway in the SaOS-2/LM7 and HOS/143B models was further validated using an orthogonal Reverse Phase Protein Array platform.

**Conclusions:**

In this study, we used a systems biology approach by integrating genomic and proteomic data to identify key and common metastatic mechanisms in OS. The use of the topological analysis revealed hidden biological pathways that are known to play critical roles in metastasis. Wnt signaling has been previously implicated in OS and other tumors, and inhibitors of Wnt signaling pathways are available for clinical testing. Further characterization of this common pathway and other topological pathways identified from this study may lead to a novel therapeutic strategy for the treatment of metastatic OS.

## Background

Osteosarcoma (OS) is the most common malignant bone tumor in children and adolescents, and accounts for approximately 60% of bone cancers in the first two decades of life [1]. Despite intensive research in the pathogenesis of this cancer, the outcome of OS patients has not significantly improved over the past three decades. The main reason for the lack of survival improvement is that this cancer is highly prone to metastasis, which is the most consistent indicator of poor outcome. Even with the most current multi-drug treatment protocols, the survival rate for patients with metastatic disease at diagnosis is only about 20% [2,3].

Similar to other types of cancer, metastasis in OS is a complex process [4,5] and a single-level of analysis alone, such as mRNA expression profiling, cannot capture the complete information to fully understand the metastatic mechanism. Therefore, a systems biology approach that takes into account different data sources such as gene and protein expression profiles is more likely to identify the dysfunctional molecules and pathways of cancer biology. Nonetheless, previous studies have revealed that direct comparisons of transcriptomic and proteomic data are difficult [6-8]. Major sources of discordance between the two types of omic data exist, such as mRNA degradation, alternative splicing, translational regulation, post-translational modifications, and protein degradation. This suggests that a new analytical approach is needed to identify biological pathways that are hidden from the direct analyses but commonly supported by various data sources. The goal of our study is to test if we could identify common metastatic processes or pathways that are jointly supported by both mRNA and protein profiling data in OS using a topological significance analysis to identify the hidden nodes.

Due to the low incidence and limited amounts of biopsy materials, clinical OS samples available for research are particularly scarce, making it extremely difficult to analyze the tumor samples using multiple genomic and proteomic platforms [9]. To circumvent this problem, isogenic metastatic cell lines have been developed for OS research. However, many of these cell lines have different genetic origins and have not been systematically characterized at the genomic or proteomic level [10-14]. Therefore, in this study we applied a systems biology approach to analyze, integrate, and identify hidden common functional pathways from two commonly used human metastatic OS cell lines and their parental non-metastatic lines. The two human metastatic OS cell line models were HOS/143B and SaOS-2/LM7. The HOS cell line, originally known as M.T. and later as TE-85, was derived from an OS of a 13 year-old girl. The 143B metastatic subline was generated from HOS by a Ki-RAS oncogene transformation [15]. On the other hand, the SaOS-2 cell was derived from an OS of an 11 year-old girl, and its metastatic subline LM7, was developed by multiple *in vivo* selection of SaOS-2 cells in mice with pulmonary metastases [14,16]. Our systems biology approach involved the analysis of the transcriptomes and glycoproteomes of the two pairs of cell lines. We validated our results using Western blotting and Reverse Phase Protein Arrays (RPPA), and the clinical significance of our findings are discussed.

## Results

To understand the common dysregulated processes in metastatic OS, we employed a systems biology approach to characterize the transcriptomes and proteomes of two commonly used human metastatic OS cell line models. The two models used were HOS and SaOS-2 and their isogenic metastatic sublines, 143B and LM7 respectively [14,15].

### mRNA expression data

mRNA expression microarray analysis was performed on both models, and the differentially expressed genes within each cell line model were identified by comparing the metastatic versus non-metastatic cell lines. The differentially expressed genes were identified using two criteria: a p-value of differential expression less than 0.05 and a fold change between the two classes greater than 2-fold. We identified 1,576 up-regulated genes and 1,656 down-regulated genes in the 143B cell relative to the HOS cell, and 648 up-regulated genes and 745 down-regulated genes in the LM7 cell relative to the SaOS-2 cell (See Tables 1 and 2). The two models had 102 common features in the up-regulated gene profiles and 157 of the down-regulated genes. By comparing to the LM7/SaOS-2 results, which constitute smaller numbers of differentially expressed genes, the common features represent 16% of up-regulated genes and 21% of down-regulated genes. The overall level of concordance for all differentially regulated genes between the two models was 19% of the differentially expressed genes in the LM7/SaOS-2 model.

**Table 1:** Top 10 up-regulated genes in HOS/143B model

**Up-regulated genes**	**Entrez Gene**	**HOS/143B (Log2)**	**p-value**	**Function**
FOXR1	283150	8	8.4 x 10E-6	Play roles in determining cell fates during early development
RTN1	6252	7	1.1 x 10E-5	Involved in membrane trafficking in neuroendocrine cells
FRMD3	257019	7	1.7 x 10E-5	Structural protein essential for erythrocyte shape and mechanical properties
HOXA9	3205	7	1.4 x 10E-5	Transcription factor which may regulate gene expression, morphogenesis and differentiation
ANGPT2	285	7	9.4 x 10E-6	In concert with VEGF, may serve as a permissive angiogenic signal
KRT18	3875	6	1.1 x 10E-5	Involved in the uptake of thrombin-antithrombin complexes by hepatic cells
IL10RA	3587	6	1.0 x 10E-5	Interferon receptor that mediates the immunosuppressive signal of IL10
TPD52	7163	6	2.0 x 10E-5	Uncharacterized function, but expressed in many tumor cell lines including colon, breast, prostate, pancreas and kidney
CA2	760	6	1.8 x 10E-5	Essential for bone resorption and osteoclast differentiation
BEND4	389206	6	1.2 x 10E-5	Uncharacterized function, but known to be phosphorylated upon DNA damage

**Table 2:** Top 10 up-regulated genes in SaOS-2/LM7 model

**Up-regulated genes**	**Entrez Gene**	**SaOS-2/LM7 (Log2)**	**p-value**	**Function**
NES	10763	30	1.8 x 10E-11	May play a role in the trafficking and distribution of intermediate filaments and other cell factors during cell division
WDR72	256764	25	1.8 x 10E-9	Involved in enamel formation
VAMP8	8673	24	1.5 x 10E-10	Involved in the targeting and fusion of transport vesicles to their target membrane
MUC15	143662	23	3.2 x 10E-12	May play a role in the cell adhesion to the extracellular matrix
LPPR4	9890	21	1.5 x 10E-10	Facilitates axonal outgrowth during development and regenerative sprouting
PTPRB	5787	18	1.9 x 10E-9	Plays an important role in blood vessel remodelling and angiogenesis
WIF1	11197	18	5.6 x 10E-11	Binds to Wnt proteins and inhibits their activities
PTPRR	5801	17	4.6 x 10E-9	Sequesters protein kinases in the cytoplasm in an inactive form and releases them for activation and translocation into the nucleus
FIBIN	387758	17	1.5 x 10E-11	Uncharacterized
SYK	6850	16	1.0 x 10E-10	Regulates several processes including immunity, cell adhesion, osteoclast maturation, platelet activation and vascular development

**Figure 1:**
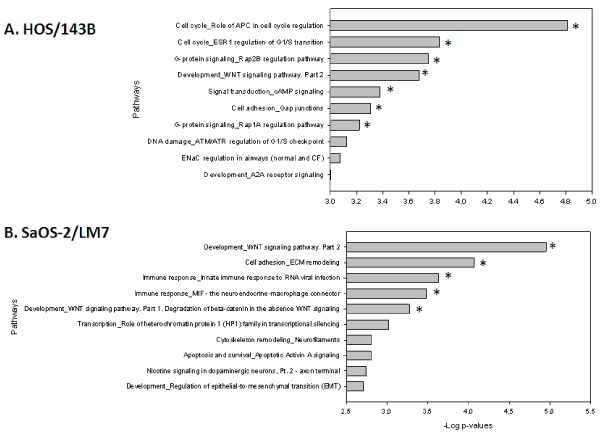
**Pathway analysis of up-regulated genes from HOS/143B and SaOS-2/LM7 models.** Top 10 significant pathways identified by MetaCore using all the significantly up-regulated genes from **(A)** HOS/143B and **(b)** SaOS-2/LM7 models. Only one common pathway, the “Development_WNT signaling pathway. Part 2”, was identified in the top 10 significant pathways between the two models. Bars quantify the –log (p-value) indicating the enrichment of the individual pathways identified. False discovery rate (FDR) is calculated for all identified pathways and a cutoff of FDR < 0.05 is set to define pathways as significant. Asterisks denote significant pathways shown.

To identify the pathways that are enriched in the mRNA expression data, the differentially regulated genes from the cell line models were analyzed by calculating enrichment in functional pathways. The most significant pathways from the HOS/143B model were associated with important biological functions such as cell cycle regulation and G-protein signaling in the up-regulated genes, and cell adhesion and immune response in the down-regulated genes. For the SaOS-2/LM7 model, the most significant pathways were associated with development and cell adhesion in the up-regulated genes, and muscle contraction and cytoskeleton remodeling in the down-regulated genes. By analyzing all significant pathways, we found only one common pathway, the “Development_WNT signaling pathway”, in the up-regulated genes between the two models (Figure 1). Similarly, there were 3 common pathways in the down-regulated genes, namely the “Muscle contraction_GPCRs in the regulation of smooth muscle tone”, “Cytoskeleton remodeling_Regulation of actin cytoskeleton by Rho GTPases”, and “Development_MAG-dependent inhibition of neurite outgrowth” (See Additional file 1: Table S 1).

### Glycogenes expression and pathway analysis

Since there is no single platform that can characterize the whole proteome in the cell and many of the clinically applicable tumor biomarkers, such as Prostate-Specific Antigen (PSA) and Carcinoembryonic Antigen (CA125), are glycoproteins [17,18], we tested if glycosylation was aberrantly regulated in the human metastatic cell line models. The mRNA expression data of 191 known human glycogenes in the metastatic and non-metastatic cell lines were investigated. This panel of glycogenes represents a comprehensive group of known human genes related to glycosylation and glycan synthesis pathways [19]. The analysis showed that 23 and 9 glycogenes had a p-value of differential expression less than 0.05 and a fold change greater than 2-fold in the HOS/143B and SaOS-2/LM7 models, respectively. Pathway analysis using all 191 glycogenes identified the baseline pathways involved in these glycogenes. The top significant baseline pathways are O-glycan biosynthesis, neolacto-series glycosphingolipids metabolism, and Keratan sulfate metabolism (Additional file 2: Figure S 1). Then, the pathway analysis was performed on the differentially regulated glycogenes from both cell line models and the results were compared to the baseline glyco-pathways. Results showed that “N-Glycan biosynthesis” was the top common pathway between the two models (Additional file 3: Figure S 2). These results suggest that the N-linked glycosylation may be involved in the metastatic process of OS. Analysis of the N-linked glycoproteome will likely provide important information in metastatic OS.

### Glycoproteomic analysis by a lectin column followed by multidimensional protein identification technology (MudPIT)

Next, we used an affinity-based mass spectrometry approach to identify the differentially expressed glycoproteins in the two cell line models. First, we used wheat germ agglutinin (WGA) lectin chromatography to selectively capture N-linked glycoproteins (Additional file 4). Subsequently, MudPIT analysis was performed on the captured glycoproteins from the two pairs of human metastatic OS cell line models. Totally, 14,928 spectra were detected in the HOS/143B model, and 34,834 spectra in the SaOS-2/LM7 model. These spectra corresponded to 3,147 and 3,123 unique peptides, which gave rise to 290 and 260 unique proteins identified in the HOS/143B and SaOS-2/LM7 models, respectively. The protein identification criteria were (1) at least two unique peptides, and (2) higher than 99% confidence of protein identification.

To measure the expressions or abundances of the glycoproteins in the metastatic OS cell line models, a spectral counting method was employed (see Methods). Spectral counting entails averaging the spectrum counts across the different protein samples tested, followed by calculating the quantitative value for each identified protein. The quantitative values of the identified proteins in the metastatic versus non-metastatic cell lines were then compared and their respective fold change was calculated. Then, the p-value for the differential expression of each protein between the two classes was calculated by Fisher’s exact test [20] and corrected for multiple testing using a step-up method [21]. Proteins with a corrected p-value less than 0.05 and a greater than 2-fold expression were considered significantly differentially expressed. The analysis showed that 90 and 135 glycoproteins were differentially regulated in the HOS/143B and SaOS-2/LM7 models, respectively. 54 glycoproteins were up-regulated and 36 glycoproteins down-regulated in the HOS/143B model (Table 3); and 75 glycoproteins were up-regulated and 60 glycoproteins were down-regulated in the SaOS-2/LM7 model (Table 4). The models had 6 common proteins in the up-regulated glycoprotein profiles and 2 common proteins in the down-regulated glycoprotein profiles. By comparing to the results of LM7/SaOs-2, the common differentially expressed proteins represent 11% of up-regulated glycoproteins and 6% of down-regulated glycoproteins. The overall level of concordance for all differentially regulated glycoproteins between the two models was 9% of the differently expressed glycoproteins in the LM7/SaOS-2 model.

**Table 3:** Top 10 up-regulated glycoproteins in HOS/143B model

**Gene Symbol**	**Accession #**	**HOS/143B**	**p-value**	**Unique Peptides**	**Function**
CANX (cDNA FLJ55574)	IPI00020984	32	1.8 x 10E-9	7	Molecular chaperone and protein translation quality control machinery
RPSAP55	IPI00399077	32	1.8 x 10E-9	2	Uncharacterized
YWHAQ	IPI00018146	32	1.8 x 10E-9	2	Regulation of a large spectrum of signaling pathway
ENO1	IPI00465248	25	3.2 x 10E-7	8	Role in glycolysis, growth control, and hypoxia tolerance
LDHB	IPI00219217	23	9.2 x 10E-7	9	Catalyzes the interconversion of pyruvate and lactate
EIF4A3	IPI00009328	23	9.2 x 10E-7	2	ATP-dependent RNA helicase
GAPDH	IPI00219018	29	9.4 x 10E-9	9	Role in glycolysis, transcription, RNA transport, DNA replication and apoptosis
EIF3	IPI00012795	18	2.5 x 10E-5	4	Required for several steps in the initiation of protein synthesis
GLUD1	IPI00016801	16	7.7 x 10E-5	5	Deamination of glutamate and role in energy homeostasis
PSME1	IPI00479722	16	7.7 x 10E-5	4	Immunoproteasome assembly and efficient antigen processing

**Table 4:** Top 10 up-regulated glycoproteins in SaOS-2/LM7 model

**Gene** **Symbol**	**Accession #**	**LM7/** **SaOS-2**	**p-value**	**Unique Peptides**	**Function**
VIM	IPI00418471	350	1 x 10E-42	22	Filaments expressed in neoplasms originated from mesenchymal cells
TPM1	IPI00940084	130	2.2 x 10E-41	2	Contractile system of muscles and the cytoskeleton of non-muscle cells
ALB	IPI00022434	120	2 x 10E-38	9	Primarily a carrier protein in serum
ODZ3	IPI00398020	97	1.1 x 10E-30	2	May function as signal transducer
KRT5	IPI00009867	90	1.4 x 10E-28	2	Filaments found in basal layer of epidermis
WDR49	IPI00216853	86	2.7 x 10E-27	2	Regulates cell division, cell-fate determination, gene transcription, and transmembrane signaling
A2M	IPI00478003	84	1.9 x 10E-26	6	Inhibits all classes of proteinases by a unique 'trapping' mechanism
NES	IPI00010800	81	1.3 x 10E-25	16	Disassembly of vimentin, role in the trafficking and distribution of cellular factors during progenitor cell division
CAL2	IPI00916600	70	3.4 x 10E-22	3	Mediates the control of numerous enzymes and proteins by calcium
ANXA5	IPI00329801	58	2.4 x 10E-18	6	Anticoagulant, inhibitor of the thromboplastin-specific complex

To show that the identified proteins were likely to be glycosylated, the top 20 up-regulated proteins from each cell line model were analyzed using a post-translational modification prediction algorithm. The selected proteins were modeled using Net-*N*-Glyc [22,23] for prediction of N-glycosylation sites in human proteins*.* The threshold was established at greater than 50% potential of glycosylation for each identified site. Results showed that 70% and 75% of the top proteins in HOS/143B and SaOS-2/LM7, respectively, have asparagines predicted to be N-glycosylated.

**Figure 2:**
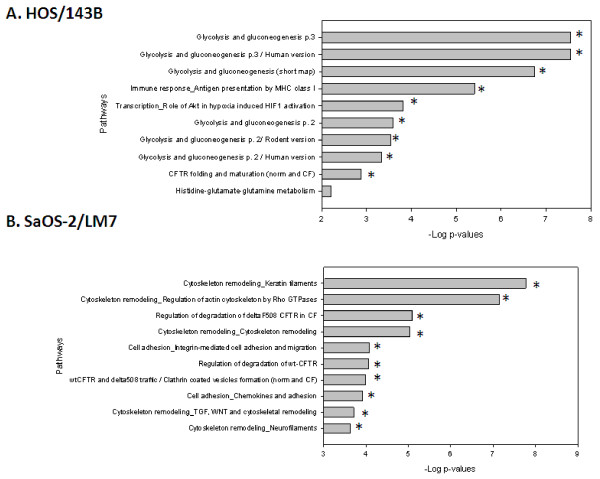
**Pathway analysis of up-regulated glycoproteins from HOS/143B and SaOS-2/LM7 models.** Top 10 significant pathways identified by MetaCore using all the significantly up-regulated glycoproteins from **(A)** HOS/143B and **(B)** SaOS-2/LM7 models. No common significant pathways were identified in the top 10 pathways between the two models. All pathways shown are significant. Refer to Figure 1 legend for graph details.

Up-regulated proteins in each model included 14-3-3 protein theta and alpha enolase from HOS/143B, and nestin and vimentin from SaOS-2/LM7, all of which have been implicated in cancer progression and metastasis [24-28]. Nonetheless, when the differentially regulated proteins were interrogated by the pathway analysis, only one common significant pathway, the “Immune response_Antigen presentation by MHC class I”, was found in the up-regulated proteins (Figure 2). In the down-regulated proteins there were 3 common significant pathways, namely the “Proteolysis_Role of Parikin in the Ubiquitin-Proteasomal Pathway”, “Cytoskeleton remodeling_Keratin filaments”, and “Regulation of CFTR activity”. Notably, none of these pathways was common at both the mRNA and protein levels in the two models.

### Validation of the differentially expressed glycoproteins by Western blot

**Figure 3:**
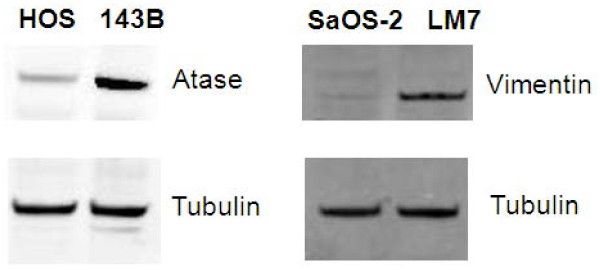
**Western blot validation of atase and vimentin.** The glycoproteomic analysis identified atase and vimentin to be significantly up-regulated in metastatic 143B and LM7 cell lines, respectively. ß-Tubulin was used as a loading control (second row)

Two candidate glycoproteins were selected for validation based on criteria that required the candidates to be among the top significantly up-regulated glycoproteins from MudPIT, be supported by the transcriptomic data, and have a commercially available antibody for validation experiments. The first candidate glycoprotein selected for validation was amidophosphoribosyltransferase (Atase), which was up-regulated by 14-fold in the metastatic 143B relative to the non-metastatic HOS in the MudPIT analysis. The other candidate was vimentin, which was up-regulated by 350-fold in the metastatic LM7 relative to the non-metastatic SaOS-2 in the MudPIT analysis. The results confirmed that both proteins were overexpressed in the metastatic sublines compared to the parental cell line (Figure 3).

### Topological significance scoring analysis of genomic and glycoproteomic data

Although the direct analyses indicated that no dysregulated pathways were common between the two cell line models at both the mRNA and protein levels, we hypothesized that common metastatic processes could be uncovered by identifying the hidden nodes in the data. We applied the “shortest-path” algorithm of topological scoring to the differentially regulated genes and glycoproteins. This algorithm has been shown previously to be able to identify significant topological nodes with respect to the differentially expressed genes or proteins by combining the high-throughput data with the global network of protein interactions [29]. These significant nodes represent a common set of signaling proteins responsible for changes in the expression of target genes and proteins. The activity of such nodes often remains hidden from direct genomic and proteomic analyses due to posttranslational modifications, binding to second messengers, recruitment to sub-cellular compartments, or specific limitations of the approach used, such as glycoproteomics focusing on a specific subproteome [29,30]. In our study, the identified hidden nodes may reveal common dysregulated pathways between the two models at the genomic and proteomic levels associated to the metastatic phenotype.

In the HOS/143B model, the topological analysis revealed 663 and 1,434 significant nodes for the up-regulated genes and down-regulated genes respectively, and 242 and 325 nodes for the up-regulated proteins and down-regulated proteins respectively. For the SaOS-2/LM7 model, there are 474 and 450 significant nodes for the up-regulated genes and down-regulated genes respectively, and 424 and 180 significant nodes for up-regulated proteins and down-regulated proteins respectively. The numbers of common genes between the models for the gene topological profiles were 85 for up-regulated genes and 199 for down-regulated genes. For the protein topological sets, there are 50 common proteins for the up-regulated proteins and 27 for the down-regulated proteins. When compared to the direct analyses, the level of concordance in the differentially regulated molecules between the two models increased in both, the genomic profiles (from 19% to 31%) and proteomic profiles (from 9% to 18%) following the topological analysis.

Additionally, the majority of the topological significant nodes identified were hidden molecules that were not present in the differentially regulated genes and proteins sets. In the transcriptomic profiles, 85% and 95% of the topological nodes from HOS/143B and LM7/SaOS2 respectively, represent hidden molecules that were not identified by the direct mRNA analysis. Similarly, in the proteomic profiles 93% and 91% of the topological nodes represent hidden molecules in the HOS/143B and LM7/SaOS2, respectively. Significant topological nodes that had been already identified by the direct transcriptomic analysis, and were therefore not hidden, include molecules such as chemokine (CXCL2), oncogenes suppressor (TP53), growth factor receptor (IGF1R), and downstream Wnt signaling factor (JUN). Conversely, topological nodes that were not hidden to the direct proteomic analysis include factors involved in cell division cycle (CDC37), eukaryotic translation elongation (EEF1A1), and interleukin enhancement (ILF2, ILF3).

**Figure 4:**
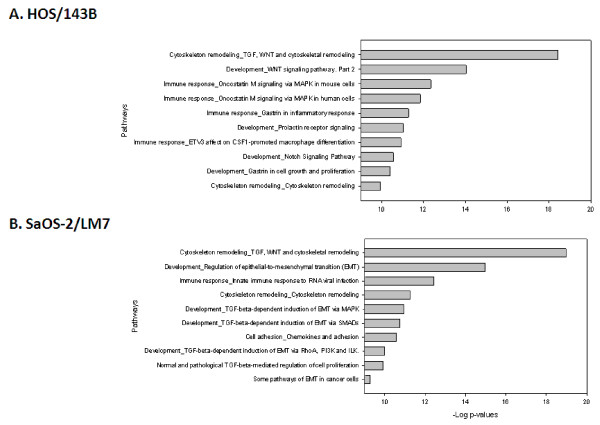
**Topological nodes of up-regulated genes from HOS/143B and SaOS-2/LM7 models**. Top 10 significant pathways identified by MetaCore using all the topological significant nodes from the up-regulated genes from **(A)** HOS/143B and **(B)** SaOS-2/LM7 models. Two common significant pathways were identified in the top 10 pathways of the two models, namely the “Cytoskeleton remodeling/TGF/WNT” and the “Cytoskeleton remodeling/Cytoskeleton remodeling”. All pathways shown are significant. Refer to Figure 1 legend for graph details.

**Figure 5:**
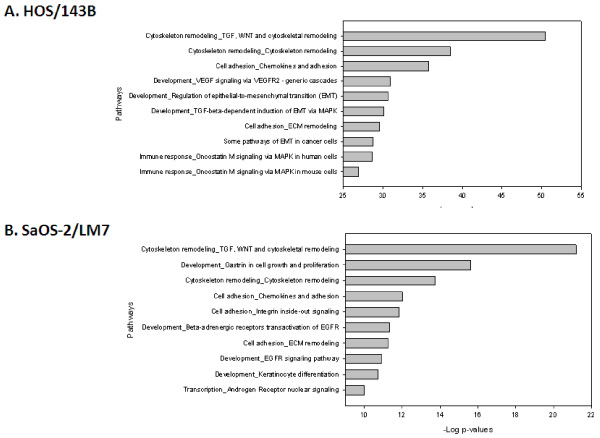
**Topological nodes of down-regulated genes from HOS/143B and SaOS-2/LM7 models.** Top significant pathways identified by MetaCore using all the topological significant nodes from the down-regulated genes from **(A)** HOS/143B and **(B)** SaOS-2/LM7 models. Four common significant pathways were identified in the top 10 pathways of the two models, namely the “Cytoskeleton remodeling/TGF/WNT”, “Cytoskeleton remodeling/Cytoskeleton remodeling”, “Cell adhesion_Chemokines and adhesion”, and “Cell adhesion_ECM remodeling”. All pathways shown are significant. Refer to Figure 1 legend for graph details.

**Figure 6:**
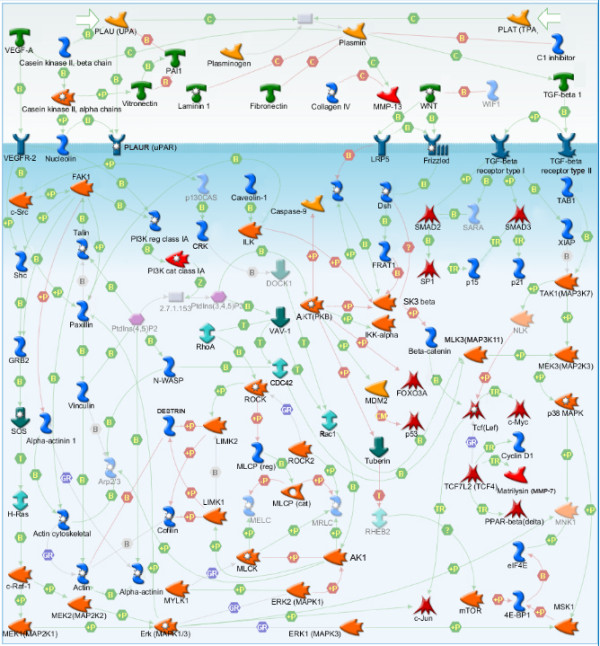
**Map of Cytoskeleton remodeling/TGF/WNT pathway showing the molecules identified by the topological significance analysis using significantly up-regulated genes and up-regulated proteins from the two metastatic models.** The highlighted molecules in the pathway represent the molecules identified by the topological analysis of the genomic and/or proteomic datasets.

On the other hand, the topological nodes that remained hidden to the direct transcriptomic and proteomic analyses include a much larger number of molecules, many of which also belong to important cancer associated families such as SMAD (SMAD1, 2, 3, 4, 7), FOX (FOXA2, C1, C2, M1), GATA (GATA1, 2, 3, 6), and MMP (MMP7, 8, 10, 13, 16, 20). These hidden nodes may carry additional information of functional processes and pathways that were unidentifiable in the direct analyses of differentially regulated genes and glycoproteins. Therefore, after the statistically significant nodes were identified, they were further interrogated to identify the associated pathways. In contrast to the previous pathway analysis results, the topological nodes analysis revealed a remarkable concordance between the two metastatic models. In the top 10 significant pathways of the genomic data, we identified that 2 and 4 pathways were common between the 2 models from the topological analysis of up-regulated genes and down-regulated genes, respectively (Figure 4 and Figure 5). The common pathways from the differentially regulated genes are the “Cytoskeleton remodeling/TGF/WNT”, “Cytoskeleton remodeling/Cytoskeleton remodeling”, “Cell adhesion/chemokines and adhesion”, and “Cell adhesion/ECM remodeling”. For the proteomic data, the two models shared 3 common pathways for the up-regulated and none in the down-regulated proteins in the top 10 most significant pathways. The common pathways from the differentially regulated glycoproteins nodes are the “Cytoskeleton remodeling/TGF/WNT”, “Cytoskeleton remodeling/Cytoskeleton remodeling”, and “Cell adhesion/chemokines and adhesion”. Together, we identified the “Cytoskeleton remodeling/TGF/WNT” pathway to be the most significant common pathway of the topological analysis of the up-regulated mRNA and glycoprotein profiles from both cell line models (Figure 6).

To test the specificity of our pathway analysis and if the common pathways could be identified by a random chance, we created 10 sets of 300 randomly selected genes (not differentially expressed). The number of selected genes was chosen based on the topological proteomic analysis, where approximately 300 significant molecules were identified. We then analyzed these gene sets in an identical fashion using the topological scoring algorithm and the pathway analysis. If the topological significance analysis or the pathway analysis would bias certain pathways that are not biologically relevant to the dataset, these specific pathways would be frequently identified from the analysis of randomly selected genes. The result showed that the false discovery rate (FDR) of identifying the “Cytoskeleton remodeling/TGF/WNT” within the most significant pathways was 10% if the top 10 pathways were considered (Additional file Additional file 5: Figure S3). Similarly, the FDR within the top ten pathways for the “Cytoskeleton remodeling/Cytoskeleton remodeling” and the “Cell adhesion/Chemokines and adhesion” pathways were 10% and 20%, respectively.

### Validation of the Cytoskeleton/TGF/WNT pathway using RPPA

As previously described, the Cytoskeleton/TGF/WNT pathway was the top common pathways for the topological analysis of the up-regulated genes and up-regulated glycoproteins. We used an orthogonal platform, RPPA, to validate if the proteins involved in the “Cytoskeleton remodeling/TGF/WNT” pathway were up-regulated when compared to proteins involved in other pathways. Protein lysates of the two models were analyzed by the RPPA, which consists of antibodies for 120 unique proteins. The numbers of up-regulated proteins in the RPPA were 35 and 14 for HOS/143B and SaOS-2/LM7, respectively. Of the unique proteins in the RPPA, 24 belonged to the “Cytoskeleton remodeling/TGF/WNT” pathway 16 of these 24 proteins were identified by the topological analyses of up-regulated genes and proteins. The probability of these “Cytoskeleton remodeling/TGF/WNT” pathway proteins to be up-regulated relative to all 120 proteins in the RPPA was found to be higher than expected in both, HOS/143B (45.8% vs. 29.1%) and SaOS-2/LM7 (25.0% vs. 11.7%) models, with p-values of 0.042 and 0.034, respectively (See Additional file 1: Table S 2a and S2b).

## Discussion

Although cancer is a heterogeneous disease, accumulating evidence supports the idea that signals for malignant transformation, progression and invasion are likely affected by a multitude of signaling pathways converging on several key regulators [29,31,32]. Identifying these key regulators will be critical for the development of novel cancer therapeutics. Nonetheless, due to diverse genetic backgrounds of tumors and the intrinsic limitations of high-throughput profiling technologies, many of these key regulators remain hidden from conventional single-level or direct analyses. In this study, we used a systems biology approach to reveal and identify topologically significant nodes by integrating transcriptomic and glycoproteomic data of two human metastatic OS models.

Direct pathway analyses of the mRNA and glycoproteomic profiles revealed a low concordance of differentially expressed genes or proteins and their respective functional pathways between the models. These results may in parts reflect the unique methodologies used to develop the cell line models. The metastatic 143B subline was generated *in vitro* via a Ki-RAS oncogene transformation of the HOS cell line [15], while the LM7 subline was developed *in vivo* through successive cycles of pulmonary metastases selection and re-injections in mice [14]. The different genetic background of the parental cell lines and the *in vitro* versus *in vivo* development of the respective metastatic sublines, may account for the differences observed in the genomic and glycoproteomic analysis of the models. The discordance between the mRNA and proteomics profiles was not surprising because of their known limited correlation [7,8], and the specific subproteome characterized in this study, namely the N-linked glycoproteins with affinity for WGA lectin. These fundamental differences and the large number of discordant pathways between the cell line models, increase the difficulty of prioritizing and identifying the key common regulators in the metastatic process of OS.

Despite of the different origins, the two OS cell line models share the same metastatic phenotype. Thus, we hypothesized that some key metastatic pathways are common to different tumors of the same cancer type, but remain hidden from the direct genomic and proteomic profiling analyses. Therefore, we performed further analysis and data integration to identify key nodes in the pathways that will reveal the critical players in common metastatic processes. For this analysis, we applied a recently developed “topological scoring” algorithm to integrate the genomic and glycoproteomic profiles and identified significant hidden common pathways between the two OS models. The “topological scoring” algorithm scores nodes in a network built from the experimentally derived, condition-specific genomic and proteomic profiles [29,30]. The output provides a series of signaling proteins associated with the metastatic phenotype and helps delineate the underlying biological processes. One of the main advantages of the topological algorithm is that it assesses the relative contribution of every node in the condition-specific network under study relative to its role in the global network. Thus, the “hubs” that are commonly present in different pathways and have high connectivity in the global network, are penalized if they do not have any special role related to the experimentally derived set of differentially expressed genes or proteins. On the other hand, nodes that provide significant connectivity among the differentially expressed genes or proteins are highly scored regardless of their global network interactions, rendering the results more specific.

Pathway analysis of the identified topological nodes revealed many common and significant dysregulated pathways between the models, which were missed by the direct analyses. Remarkably, the analysis revealed pathways such as “Cytoskeleton remodeling/TGF/WNT” and “Cell adhesion/chemokines and adhesion”, which were common to both models and present at both mRNA and protein levels. All the processes represented in these common pathways have been previously associated with metastasis in various cancer types, and changes involved in these signaling networks associated to cytoskeleton remodeling, cell-cell and cell-matrix adhesion are critical for the cancer cell migration and invasion [4,33-36]. To determine if these pathways would be identified by a random chance or nonspecifically, we performed a false discovery analysis using randomly selected genes and confirmed the specificity of these pathways in our datasets.

Our results support the notion that there are common metastatic mechanisms acting downstream of different genetic aberrations or origins. This implies the existence of common molecular therapeutic targets and disease biomarkers irrespective of the driver genetic abnormalities in the tumors. This finding is particularly significant in tumors, such as OS, in which the genomic abnormalities are known to be highly complex and it is hard to determine the driver mutations from the passengers [37,38]. For instance, the most significantly overrepresented pathway of the topological analysis was the “Cytoskeleton remodeling/TGF/WNT”, which includes the interesting Wnt component. The Wnt signaling is particularly important for cancer cells as they are able to disrupt this pathway in different ways resulting in tumorigenesis and metastasis. Activation of the Wnt signaling pathway is necessary for the commitment of mesenchymal stem cells to the osteoblast lineage. In addition, aberrant Wnt signaling activity has been reported in a variety of human cancers including soft tissue sarcomas and human OS primary tissues and cell lines [36,39-42]. Abnormal activation of the canonical Wnt pathway results in stabilized β-catenin that translocates into the nucleus. Subsequently, it binds to transcription factors and drives the uncontrolled expression of target genes implicated in cell proliferation, transformation, and tumor progression, such as Myc, matrix metalloproteinase 7, Axin-2, the cell adhesion molecule L1-CAM, the metastasis gene S100A4, and others [33,43,44]. Therefore, targeting the Wnt signaling may have a therapeutic effect on different types of metastatic OS.

Similarly, our findings suggest that Wnt signaling is highly relevant to the metastasis of OS. Previous studies have shown that OS harbors an accumulation of β-catenin either in the cytoplasm or in the nucleus [45], a hallmark of Wnt signaling activation. Additionally, the Wnt coreceptor LRP5 expression in OS tissue samples correlated with metastasis and a lower rate of disease-free survival in patients [46]. Furthermore, OS cell lines have been reported to express many Wnt ligands and receptors, whereas secreted Wnt antagonists including secreted frizzled-related protein (sFRP) and Dickkopf (Dkk) families are commonly absent [46,47]. Inhibition of similar mechanisms by the reintroduction of secreted Wnt antagonists in OS, such as the Wnt inhibitory factor (WIF-1), has been proposed for downregulation of Wnt signaling as a novel therapeutic approach. For instance, overexpression of WIF-1 significantly decreased tumor growth and markedly reduced the number of lung metastasis of 143B cells *in vivo*[48]. In other studies soluble LRP5 (sLRP5), which blocks Wnt signaling, was able to reduce *in vitro* cellular invasion, and transfection of sLRP5 in SaOS-2 caused a marked up-regulation of E-cadherin and down-regulation of N-cadherin suggesting a reversal of epithelial-mesenchymal transition [40]. These findings suggest an important role for aberrant Wnt signaling in the pathobiology and progression of OS. The Wnt pathway may represent a promising source of novel therapeutic targets and disease progression biomarkers.

Another common pathway relevant in metastasis is the “Cell adhesion/chemokines and adhesion”, which was identified at both the genomic and proteomic levels. Chemokine ligands and their cognate receptors have been extensively implicated in the progression and metastasis of multiple tumors such as melanoma, breast cancer, prostate cancer, and others [49-53]. In OS, research has revealed a complex interaction between chemokine ligand/receptor axis, and their role in tumor invasion, metastasis and patient prognosis [54]. Our group previously reported that expression of two CXC chemokines were elevated in tumor and plasma of pediatric OS patients, and their levels correlated with patient outcomes [55]. Several of the CXC chemokines were also identified by the topological analysis in this study, including CXCL12 which has been directly associated to metastasis in OS and other tumors [56]. Identification of the significant topological nodes within such complex networks could help identify the key players and delineate their roles in cell adhesion, invasion and other important metastasis functions.

In addition to the identification of therapeutic targets, identification of tumor-derived biomarkers for early detection of disease progression and metastasis is a critical component for personalized medicine. As previously noted, characterization of key hidden nodes could facilitate the identification of candidate biomarkers for metastatic OS. Because of the diversity of the human proteome and limitations of the current proteomic methods, targeting specific subproteomes that are likely to be secreted into the blood stream, such as the glycoproteome, will improve the likelihood of identifying tumor-derived circulating biomarkers. In this study, we characterized the N-linked glycoproteome due to their involvement in metastatic OS as evidenced by the glycogene analysis, and their frequent localization in the cellular membrane and extracellular space. In spite of the limited number of glycoproteins identified in this study, the topological analysis revealed common significant pathways between the differentially regulated glycoproteins and genes in both OS models. Using this analysis, we can prioritize the up-regulated glycoproteins and up-regulated genes as well as the hidden molecules that may serve as biomarkers for the disease or metastasis in the future validation, such as proteins in the TGF beta (i.e. TGFB1, TGFBR1, TGFBR2) and MAP kinase (MAPK1, MAPK3, MAP3K1,MAP3K8) families identified by topological analysis.

Despite the encouraging results, we recognize that there are limitations in the current study. For instance, the cell line models used in our study represents a significant limitation. Although the *in vitro* models provide a convenient and renewable platform that cannot be surpassed by clinical specimens, it may not faithfully reflect the behavior of tumor cells *in vivo*. Therefore, future directions include the development of orthotopic xenograft mouse models to recapitulate and validate the results obtained in this study. In addition, the characterized N-linked glycoproteins with an affinity for the WGA lectin represent a restricted number of proteins, which is typical to this type of subproteome analysis [57,58]. This limits the number of identifiable up-regulated glycoproteins amenable for validation as candidate biomarkers. Further studies with a wider range of proteins including additional glycoproteins by different capture methods (i.e. cell surface biotin labeling, biocytin hydrazide N-linked enrichment) [59,60] and other relevant subproteomes, such as the secretome [61,62] will help to alleviate this limitation.

## Conclusions

We used a systems biology approach to identify key and common metastatic mechanisms in OS. A similar approach can be applied to other genetically distinct but phenotypically similar cell lines of other tumor types. The use of the topological analysis revealed hidden biological networks known to play a fundamental role in metastasis. The analysis will also provide new types of information in this pediatric cancer that are not identifiable by conventional single-level analyses, and present new directions for future research. Our study shows that the systems biology analysis of metastasis through different types of genomic and proteomic characterization can shed light on the key biological processes of OS invasion. Our findings have significant implications on both the development of biomarkers for disease progression and metastasis, and identification of potential targets for novel therapies. Ultimately, we believe that the results reported in this study will accelerate the development of a personalized treatment for metastatic OS patients to maximize their survival and decrease treatment toxicity.

## Methods

### Human metastatic OS cell line models

Human osteosarcoma cell lines 143B and HOS were purchased from ATCC (Manassas, VA). The SaOS-2 and LM-7 cell lines were provided by Eugenie S. Kleinerman from The University of Texas M. D. Anderson Cancer Center. The cell lines were maintained in GIBCO Minimum Essential Media supplemented with 10% fetal bovine serum at 37^o^C in 5% CO_2_, and they were tested to be mycoplasma-free. Unless otherwise indicated, all chemicals were purchased from Sigma-Aldrich (St. Louis, MO). Cell lines were validated by STR DNA fingerprinting using the AmpF_STR Identifiler kit according to manufacturer's instructions (Applied Biosystems cat 4322288). The STR profiles were compared to known ATCC fingerprints (ATCC.org), and to the Cell Line Integrated Molecular Authentication database (CLIMA) version 0.1.200808 (http://bioinformatics.istge.it/clima/) (Nucleic Acids Research 37:D925-D932 PMCID: PMC2686526). The STR profiles matched known DNA fingerprints or were unique.

### mRNA expression profiling

The TRIzol reagent (Invitrogen, San Diego, CA) was used to extract RNA from the cell lines, and the RNA was further purified by the RNeasy kit (QIAGEN, Valencia, CA). 250ng of purified RNA were labelled by the Genechip 3” IVT express kit (Affymetrix, Santa Clara, CA) following the manufacturer’s protocol. Then the labelled RNA was hybridized with the Human U133 plus 2 array using the GeneChip hybridization, wash and stain kit (Affymetrix). The hybridization signals were obtained from the GeneChip 7G Scanner (Affymetrix).

The data was normalized using the Robust Multichip Average (RMA). RMA consists of three steps: a background adjustment, quantile normalization and summarization [63-65]. The quality of the arrays data was assessed through affyQCReport [66]. The microarray data was then analyzed using the S-score algorithm, which is a comparative method for gene expression data analysis that uses probe level data. The algorithm consists of an error model for the expression of probe pair signals in which the detected signal is assumed to be proportional to the probe pair signal for highly expressed genes, while approaching a background noise level for genes with low levels of expression. These probe pairs level data are used to calculate the significance score (S-score), which is a measure of relative change. Under conditions of no differential expression between chips, the S-scores of all the genes follow a standard normal distribution. The p-values for differential expressions of genes can be then obtained from the distribution, which accounts for multiple testing [67,68]. Differential gene expression was defined as genes with a p-value less than 0.05 and a 2-fold or greater difference in normalized fluorescence intensity between the 143B (numerator) and HOS (denominator), and LM7 (numerator) and SaOS-2 (denominator) cells.

### N-linked glycoprotein capturing

Total proteins of approximately 10^8^ cells were extracted by the M-PER Mammalian Protein Extraction Reagent (Pierce. Rockford, IL). Insoluble debris was removed by centrifugation at 14,000 g for 15 min at 4°C. The WGA high affinity lectin chromatography was used to isolate glycoproteins with N-acetyl glucosamine (GlcNAC) and terminal GlcNAC structures as described in the manufacturer’s protocol (Pierce). The eluted glycoprotein concentration was determined using BCA Protein Assay (Pierce). The glycoproteins were stored at −80°C for the mass spectrometry analysis following deglycosylation. Peptide-N-glycosidase F was used for the deglycosylation of glycoproteins as described in the manufacturer’s protocol (Sigma).

### MudPIT

The MudPIT consists of two independent separation steps, namely the strong cation exchange and the reversed phase that are coupled with mass spectrometry analysis. For the nanoLC-LC-MS/MS, a microbore HPLC system (Paradigm MS4, Michrom, Auburn, CA) was used with two separate strong cation exchange (SCX) and reversed phase (RP) columns. A representative twelve step LC-LC-MS/MS analysis was performed. The elution was directly sprayed into a custom-built nanoelectrospray ionization source of a ThermoFinnigan LTQ ion trap mass spectrometer (ThermoFinnigan, San Jose, CA). Dependent data scanning was then performed by Xcalibur v-1.4 software [69]. The MS/MS spectra were collected in an information-dependent acquisition mode at 3-second intervals.

The MS analysis was performed at the Arizona Mass Spectrometry Consortium, the University of Arizona. Tandem MS spectra of peptides were analyzed with TurboSEQUEST™ v-3.1, a program that allows the correlation of experimental tandem MS data with theoretical spectra generated from known protein sequences [70]. The peak list (dta files) for the search was generated by Bioworks 3.1. All spectra were searched against the latest version of the non-redundant protein database downloaded from NCBI. The results were also validated using X!Tandem, and with Scaffold, (Proteome Software, Portland, Oregon, USA) a program that probabilistically validates these peptide identifications and derives corresponding protein probabilities using ProteinProphet [71].

Peptide identifications were accepted if they exceeded specific database search engine thresholds. Sequest identifications required at least deltaCn scores of greater than 0.08 and XCorr scores of greater than 1.8, 2.5, 3.5 for singly, doubly, triply charged peptides. X! Tandem identifications required at least -Log(Expect Scores) scores of greater than 3.0. Protein identifications were accepted if they contained at least two identified peptides. Proteins that contained similar peptides and could not be differentiated based on MS/MS analysis alone were grouped to satisfy the principles of parsimony.

Relative expression of glycoproteins between the metastatic OS cell line models was analyzed using spectral counting function within the Scaffold software. Scaffold calculates the quantitative value number by normalizing spectral counts across the experiment. The normalized spectral count for a given protein was compared between the metastatic and non-metastatic cell line samples, the minimum quantitative value was set at 1.0, and a relative fold change between the two samples was calculated. The p-value for the differential expression of each protein was calculated using a Fisher’s exact test. This test uses spectral count statistics to measure differential protein expression in pairwise experiments, and has been found to be a reliable and preferable method when two replicates are available in label-free proteomics experiments [20]. Then, the p-values were corrected by a step-up method [21]. Differential expression was then defined as proteins having both, a corrected p-value lower than 0.05 and a fold change greater than 2.

### Data analysis

For prediction of N-glycosylation, the top 20 identified proteins from each cell line model were modeled using Net-*N*-Glyc [22,23]*.* The predictions were done only on the Asn-Xaa-Ser/Thr sequons and the threshold was established at higher than 50% potential of glycosylation for each identified site.

MetaCore was used for pathway analysis of the differentially regulated genes and glycoproteins (GeneGo Inc, St. Joseph, MI). We used all proteins in the MetaCore network as the reference list for calculating enrichment p-values. For the identification of key regulatory molecules within the genomic and glycoproteomic data, the “topological scoring” method was used. This method utilizes GeneGo’s MetaBase knowledgebase containing approximately 300,000 protein-protein and protein-small molecule interactions manually extracted from the literature by expert annotators. The algorithm used for the analysis was the “shortest-paths among nodes”, the background list used includes all network nodes, and the significance level was 0.05 [29,30]. Functional analysis using MetaCore was then performed on the identified topological nodes derived from the genomic and glycoproteomic profiles to determine the most significantly enriched canonical pathways.

As the topological analysis identified close to 300 proteins for each of the proteomic profiles, we utilized 10 sets of 300 randomly selected genes from the genome to measure the false discovery rate of the results. The random genes sets were analyzed as described above. First, functional pathway analysis was performed on the randomly selected genes. Then, topological analysis was performed on the random genes, followed by functional analysis of the identified key topological nodes. The top significant pathways identified from the initial random genes and the topological nodes were compared to the respective identified pathways from the human cell line models. Specifically, the chance of identifying each of the “Cytoskeleton remodeling/TGF/WNT”, the “Cytoskeleton remodeling/Cytoskeleton remodeling”, and the “Cell adhesion/Chemokines and adhesion” pathways within the top 10 most significant pathways in the random sets was calculated to determine their false discovery rates.

### Confirmation/validation by Western blot and RPPA

For Western blotting, the proteins were first resolved by NuPAGE 4-12% Bis-Tris gel (Invitrogen) and then transferred to a PVDF membrane. The membranes were incubated with the primary antibody against the specific protein of interest, i.e. mouse anti-human atase, rabbit anti-human vimentin, and ß-tubulin antibodies. Proteins were detected by donkey anti-mouse or goat anti-rabbit secondary antibodies (LI-COR Biosciences, Lincoln, NE). ß-tubulin was used as loading controls. The fluorescence was captured by Odyssey imaging System (LI-COR Biosciences).

Proteins involved in the “Cytoskeleton remodeling/TGF/WNT” pathway were validated using RPPA. The RPPA consists of 242 antibodies that target 120 unique proteins. The antibody with the largest differential expression for each of the 120 unique proteins was selected for further analysis. 24 out of the 120 unique proteins in the RPPA including AKT1, CAV1, EIF4E, GSK2B, JUN, MAPK1, MTOR, MYC, PTK2, TP53, and VEGFA, belonged to the “Cytoskeleton remodeling /TGF/WNT” pathway. Under the null hypothesis, the 24 proteins from the “Cytoskeleton/TGF/WNT” pathway have no preferential up-regulation relative to the total set of RPPA proteins.

For RPPA profiling, serial diluted lysates were arrayed on nitrocellulose-coated slides (Grace Biolab, Bend, OR) by Aushon 2470 Arrayer (Aushon BioSystems, Billerica, MA). Each slide was probed with a validated primary antibody plus a biotin-conjugated secondary antibody. The signal obtained was amplified using a Dako Cytomation–catalyzed system (Dako) and visualized by DAB colorimetric reaction. The slides were scanned, analyzed, and quantified using a customized Microvigene software (VigeneTech Inc., Carlisle, MA) to generate spot intensity. Each dilution curve was fitted with the logistic model “Supercurve Fitting” developed by the Department of Bioinformatics and Computational Biology in MD Anderson Cancer Center (http://bioinformatics.mdanderson.org/OOMPA).

RPPA data analysis showed that the Log2 expression of the 120 unique proteins in the 143B vs. HOS model had a mean of 0.03 with a standard deviation of 0.58. For the LM7 vs. SaOS-2 model, the mean was -0.05 with a standard deviation of 0.40. Because of the limited fold changes observed in the RPPA platform, an up-regulated protein was defined as a Log2 expression higher than 0.3 (fold change higher than 1.23). The probability of a protein’s expression being greater than a pre-determined “up-regulated” threshold follows a hypergeometric distribution. In this case, the 120 unique proteins from the RPPA represent the sample size, and the 24 “Cytoskeleton remodeling/TGF/WNT” pathway proteins represent a “selection”. If the proteins involved in the “Cytoskeleton remodeling/TGF/WNT” pathway are not enriched, then the number of “up-regulated proteins” in that pathway or “selection” follows the hypergeometric distribution

(1) $$P\left(x=k\right)=\frac{\left[\text{C}\left(\text{K,k}\right)\right]\left[\text{C}\left(\text{M,m}\right)\right]}{\left[\text{C}\left(\text{N,n}\right)\right]}$$

where *P(x = k)* is the probability of a “k” number of up-regulated proteins being present in a specific Pathway.

Note: C(A,a) = A! / [(A-a)! *(a!)]

k = number of up-regulated proteins in the Pathway

K = number of up-regulated proteins in the RPPA

m = number of not up-regulated proteins in the Pathway

M = number of not up-regulated proteins in the RPPA

n = number of all proteins in the Pathway

N = number of all proteins in the RPPA

The p-value is then calculated as the cumulative distribution of the function above.

### Availability of supporting data

The microarray data described in this study have been deposited in the Gene Expression Omnibus [72] with the accession number GSE37552. The proteomic data are available in the Digital Object Identifier System with the DOI number http://dx.doi.org/10.6070/H47P8W9B.

## Competing interests

The authors declare that there are no known competing interests regarding to this study.

## Authors’ contributions

RJF carried out the cell line work, glycoprotein analysis, Western blot validation, pathway analysis, topological scoring analysis, RPPA data analysis, and prepared the manuscript. YL participated in the cell line work, protein analysis and Western blot validation. AY processed the mRNA expression data and helped with the pathway analysis. JS and PR carried out the mRNA isolation and expression microarray analysis. SL contributed to the mass spectrometry analysis. CCL participated in the study design, data analysis, and in the interpretation of results. TKM conceived of, supervised, designed and coordinated the study, and prepared the manuscript. All authors read and approved the final manuscript.

## Supplementary Material

Additional file 1 **Table S1.** Significant Pathways from the ontology enrichment of the down-regulated genes in the HOS/143B model and SaOS-2/LM7 model. **Tables S2a and S2b**. “Cytoskeleton remodeling/TGF/WNT” pathway proteins up-regulated in the RPPA on HOS/143B and SaOS-2/LM7 models. Click here for file

Additional file 2 **Figure S1.** Pathway analysis of all glycogenes. Top significant pathways identified by MetaCore using all 191 glycogenes identified in the genomic profile. All pathways shown are significant. Refer to Figure 1 legend for graph details. Click here for file

Additional file 3 **Figure S2.** Pathway analysis of differentially regulated glycogenes from 143B/HOS and LM7/SaOS-2 models. Top significant pathways identified by MetaCore using (a) differentially regulated genes from 143B/HOS model, and (b) differentially regulated genes from LM7/SaOS-2 model. Results showed that “N-Glycan biosynthesis” was the top common pathway between the two models. Dark orange bars represent significant pathways. Refer to Figure 1 legend for graph details. Click here for file

Additional file 4 Methods for the N-Linked glycoproteins enrichment by lectin affinity chromatography. Click here for file

Additional file 5 **Figure S3.** Pathway analysis of random genes and their topological nodes. Top significant pathways identified by MetaCore using (a) 300 randomly selected genes and (b) topological significant nodes from the 300 randomly selected genes. None of the top common significant pathways identified from the topological analysis of the up-regulated genes and up-regulated glycoproteins from the 143B/HOS and LM7/SaOS-2 models were identified by the topological analysis of this random gene set. Dark orange bars represent significant pathways. Refer to Figure 1 legend for graph details. Click here for file
