# Supplementary material for: A systems biology approach reveals common metastatic pathways in osteosarcoma
Source: BMC Syst Biol. 2012 May 28;6:50. doi: 10.1186/1752-0509-6-50 (PMC3431263; doi:10.1186/1752-0509-6-50)
Supplement: Additional file 1 — Table S1. Significant Pathways from the ontology enrichment of the down-regulated genes in the HOS/143B model and SaOS-2/LM7 model. Tables S2a and S2b. “Cytoskeleton remodeling/TGF/WNT” pathway proteins up-regulated in the RPPA on HOS/143B and SaOS-2/LM7 models. [file 1752-0509-6-50-S1.pdf]

Supplemental Table 1. Significant Pathways from the Ontology enrichment of the down-regulated genes in the HOS/143B model and SaOS-2/LM7 model.

**Significant Pathways from downregulated genes in HOS/143B model**	**Significant Pathways from downregulated genes in SaOS-2/LM7 model**
Cell adhesion_Chemokines and adhesion	**Muscle contraction_GPCRs in the regulation of smooth muscle tone**
Cell adhesion_ECM remodeling	**Cytoskeleton remodeling_Regulation of actin cytoskeleton by Rho GTPases**
Immune response_Oncostatin M signaling via MAPK in human cells	Cell adhesion_Tight junctions
Cytoskeleton remodeling_Cytoskeleton remodeling	Signal transduction_PKA signaling
Cytoskeleton remodeling_TGF, WNT and cytoskeletal remodeling	**Development_MAG-dependent inhibition of neurite outgrowth**
Development_Regulation of epithelial-to-mesenchymal transition (EMT)	
Cell adhesion_Integrin-mediated cell adhesion and migration	
Immune response_Oncostatin M signaling via MAPK in mouse cells	
Immune response_Oncostatin M signaling via JAK-Stat in human cells	
Cell adhesion_PLAU signaling	
Transport_Macropinocytosis regulation by growth factors	
Cell adhesion_Role of tetraspanins in the integrin-mediated cell adhesion	
Development_Beta-adrenergic receptors transactivation of EGFR	
Cell adhesion_Cell-matrix glycoconjugates	
Development_TGF-beta-dependent induction of EMT via MAPK	
Development_FGFR signaling pathway	
Immune response_Oncostatin M signaling via JAK-Stat in mouse cells	
Development_EPO-induced Jak-STAT pathway	
Development_Dopamine D2 receptor transactivation of EGFR	
Development_Slit-Robo signaling	
Development_TGF-beta receptor signaling	
Cytoskeleton remodeling_Fibronectin-binding integrins in cell motility	
Development_S1P2 and S1P3 receptors in cell proliferation and differentiation	
Development_PDGF signaling via STATs and NF-kB	
Development_TGF-beta-dependent induction of EMT via RhoA, PI3K and ILK.	
Apoptosis and survival_Caspase cascade	
Development_S1P1 receptor signaling via beta-arrestin	
Normal and pathological TGF-beta-mediated regulation of cell proliferation	
Translation_Non-genomic (rapid) action of Androgen Receptor	

Development_PIP3 signaling in cardiac myocytes
Immune response_IL-2 activation and signaling pathway
Apoptosis and survival_BAD phosphorylation
**Cytoskeleton remodeling_Regulation of actin cytoskeleton by Rho GTPases**
Immune response_IL-6 signaling pathway
Immune response_IL-9 signaling pathway
Development_Prolactin receptor signaling
**Development_MAG-dependent inhibition of neurite outgrowth**
Development_IGF-1 receptor signaling
Neurophysiological process_Receptor-mediated axon growth repulsion
Transcription_Androgen Receptor nuclear signaling
Development_Ligand-independent activation of ESR1 and ESR2
Cytoskeleton remodeling_Neurofilaments
Signal transduction_PTEN pathway
Development_ERBB-family signaling
Membrane-bound ESR1: interaction with G-proteins signaling
Immune response _IFN gamma signaling pathway
Development_Gastrin in cell growth and proliferation
Development_Alpha-2 adrenergic receptor activation of ERK
Development_FGF2-dependent induction of EMT
Development_BMP signaling
Development_EGFR signaling pathway
Immune response_IL-15 signaling
Cell adhesion_Integrin inside-out signaling
Development_Keratinocyte differentiation
Development_CNTF receptor signaling
Cytoskeleton remodeling_Integrin outside-in signaling
Cell adhesion_Plasmin signaling
Development_TGF-beta-dependent induction of EMT via SMADs
**Muscle contraction_GPCRs in the regulation of smooth muscle tone**
Development_EDNRB signaling
Development_Role of IL-8 in angiogenesis
Development_VEGF signaling via VEGFR2 - generic cascades
Development_S1P3 receptor signaling pathway
Chemotaxis_Inhibitory action of lipoxins on IL-8- and Leukotriene B4-induced neutrophil migration
Some pathways of EMT in cancer cells
Immune response_IL-17 signaling pathways
Development_FGF-family signaling
Development_Delta-type opioid receptor mediated cardioprotection

Immune response_IL-4 - antiapoptotic action
Cell adhesion_Gap junctions
Immune response_Signaling pathway mediated by IL-6 and IL-1
Cell adhesion_Histamine H1 receptor signaling in the interruption of cell barrier integrity
Immune response_Role of integrins in NK cells cytotoxicity
Proteolysis_Role of Parkin in the Ubiquitin-Proteasomal Pathway
Immune response_IFN alpha/beta signaling pathway
Cell adhesion_Endothelial cell contacts by non-junctional mechanisms
Development_Transactivation of PDGFR in non-neuronal cells by Dopamine D2 receptor
Development_G-Proteins mediated regulation MAPK-ERK signaling
Development_Endothelin-1/EDNRA transactivation of EGFR
Transport_Clathrin-coated vesicle cycle
Transcription_Receptor-mediated HIF regulation
PGE2 pathways in cancer
Transcription_CREB pathway
Apoptosis and survival_Apoptotic Activin A signaling
Development_Neurotrophin family signaling
Cytoskeleton remodeling_Alpha-1A adrenergic receptor-dependent inhibition of PI3K
Immune response_Histamine H1 receptor signaling in immune response
Development_Angiotensin activation of ERK
Development_EGFR signaling via small GTPases
Immune response_IL-22 signaling pathway
Muscle contraction_Delta-type opioid receptor in smooth muscle contraction
Inhibitory action of Lipoxins on neutrophil migration
Development_VEGF-family signaling
Development_PEDF signaling
Immune response_Role of HMGB1 in dendritic cell maturation and migration
Inhibitory action of Lipoxin A4 on PDGF, EGF and LTD4 signaling
Signal transduction_AKT signaling
Development_Mu-type opioid receptor regulation of proliferation
Development_Lipoxin inhibitory action on PDGF, EGF and LTD4 signaling
Immune response_IL-4 signaling pathway
Immune response_PGE2 in immune and neuroendocrine system interactions
Development_Flt3 signaling
Immune response_IL-1 signaling pathway
Immune response_HMGB1/RAGE signaling pathway

Cell cycle_Influence of Ras and Rho proteins on G1/S Transition
NGF activation of NF-kB
Development_Thrombopoetin signaling via JAK-STAT pathway
Development_Thrombopoietin-regulated cell processes
G-protein signaling_G-Protein alpha-12 signaling pathway
Muscle contraction_S1P2 receptor-mediated smooth muscle contraction
Development_HGF signaling pathway
Development_PDGF signaling via MAPK cascades
Immune response_IL-3 activation and signaling pathway
Immune response_CD40 signaling
Blood coagulation_Blood coagulation
Development_Mu-type opioid receptor signaling via Beta-arrestin
Cytoskeleton remodeling_FAK signaling
Apoptosis and survival_Ceramides signaling pathway
Reproduction_Progesterone-mediated oocyte maturation
Apoptosis and survival_Granzyme B signaling
Cell cycle_Role of Nek in cell cycle regulation
Signal transduction_ERK1/2 signaling pathway
Immune response _CCR3 signaling in eosinophils
Apoptosis and survival_Anti-apoptotic TNFs/NF-kB/Bcl-2 pathway
G-protein signaling_Regulation of CDC42 activity
Apoptosis and survival_HTR1A signaling
Development_GM-CSF signaling
Immune response_IL-13 signaling via PI3K-ERK
Apoptosis and survival_Apoptotic TNF-family pathways
Immune response_IL-10 signaling pathway
Signal transduction_Erk Interactions: Inhibition of Erk
Chemotaxis_CXCR4 signaling pathway
Apoptosis and survival_Role of CDK5 in neuronal death and survival
Regulation of lipid metabolism_Insulin regulation of fatty acid methabolism
Regulation of lipid metabolism_Regulation of fatty acid synthase activity in hepatocytes
Development_ACM2 and ACM4 activation of ERK
Immune response_HTR2A-induced activation of cPLA2
Transcription_Role of VDR in regulation of genes involved in osteoporosis
Transport_Intracellular cholesterol transport in norm
Immune response_PGE2 common pathways
Blood coagulation_GPCRs in platelet aggregation
G-protein signaling_S1P2 receptor signaling
Development_Angiopoietin - Tie2 signaling

Development_S1P1 signaling pathway
Translation _Regulation of EIF4F activity
Cytoskeleton remodeling_Keratin filaments
Development_Activation of ERK by Kappa-type opioid receptor
Development_Thrombospondin-1 signaling
Development_Membrane-bound ESR1: interaction with growth factors signaling
Role of alpha-6/beta-4 integrins in carcinoma progression
Signal transduction_Calcium signaling
Regulation of metabolism_Bile acids regulation of glucose and lipid metabolism via FXR
Development_GDNF family signaling
Chemotaxis_Lipoxin inhibitory action on fMLP-induced neutrophil chemotaxis
Glutathione metabolism

Supplemental Table 1 Legend.

Supplemental Table 1 shows all the significant pathways identified by MetaCore using all the significantly down-regulated genes from HOS/143B and SaOS-2/LM7 models. False discovery rate was calculated for all identified pathways and a cutoff of FDR < 0.05 was set to define pathways as significant. The 3 common pathways between the two models are highlighted in bold.

Supplemental Tables 2a and 2b. Cytoskeleton/TGF/WNT Pathway proteins up-regulated in the RPPA on HOS/143B model and SaOS-2/LM7.

Supplemental Table 2a. Cytoskeleton/TGF/WNT Pathway proteins up-regulated in the RPPA on HOS/143B model. Out of 120 proteins in the RPPA, 35 were up-regulated in HOS/143B model. 11 of the up-regulated proteins belong to the Cytoskeleton/TGF/WNT Pathway

Gene Symbol	Accession #	Log 2	Function
CTNNB1	IPI00017292	0.87	Key downstream component of the canonical Wnt signaling pathway involved in the regulation of cell adhesion
PTK2	IPI00793270	0.76	Implicated in signaling pathways involved in cell motility, proliferation and apoptosis
TP53	IPI00025087	0.72	Acts as a tumor suppressor in many tumor types; induces growth arrest or apoptosis
CCND1	IPI00028098	0.60	Regulatory component that inhibits members of the retinoblastoma protein family and regulates the cell-cycle
MAP2K1	IPI00219604	0.57	Mediates diverse functions such as cell growth, adhesion, survival and differentiation
XIAP	IPI00303890	0.52	Multiple functions including regulation of apoptosis and inflammatory signaling, copper homeostasis, mitogenic kinase signaling, cell proliferation, cell invasion and metastasis
MTOR	IPI00031410	0.35	Central regulator of cellular metabolism, growth and survival in response to hormones, growth factors, nutrients, energy and stress signals
AKT1	IPI00012866	0.34	Regulate many processes including metabolism, proliferation, cell survival, growth and angiogenesis.
MAPK1	IPI00003479	0.31	Mediates diverse biological functions such as cell growth, adhesion, survival and differentiation
PXN	IPI00220030	0.30	Cytoskeletal protein involved in actin-membrane attachment at sites of cell adhesion to the extracellular matrix
JUN	IPI00008965	0.30	Transcription factor that interacts directly with specific target DNA sequences to regulate gene expression (nhancer heptamer motif 5'-TGA[CG]TCA-3')

Supplemental Table 2b. Cytoskeleton/TGF/WNT Pathway proteins up-regulated in the RPPA on SaOS-2/LM7 model. Out of 120 proteins in the RPPA, 14 were up-regulated in the SaOS-2/LM7 model. 6 of the up-regulated proteins belong to the Cytoskeleton/TGF/WNT Pathway

Gene Symbol	Accession #	Log 2	Function
AKT1	IPI00012866	1.02	Regulate many processes including metabolism, proliferation, cell survival, growth and angiogenesis
FN1	IPI00022418	0.74	Involved in cell adhesion, cell motility, opsonization, wound healing, and maintenance of cell shape
PXN	IPI00220030	0.34	Cytoskeletal protein involved in actin-membrane attachment at sites of cell adhesion to the extracellular matrix
MTOR	IPI00031410	0.34	Central regulator of cellular metabolism, growth and survival in response to hormones, growth factors, nutrients, energy and stress signals
CAV1	IPI00009236	0.33	May act as a scaffolding protein within caveolar membranes, and regulate G-protein alpha subunits activity
MAPK14	IPI00002857	0.30	Important role in cellular responses evoked by extracellular stimuli such leading to activation of transcription factors
