# Supplementary figures and images for: A systems biology approach reveals common metastatic pathways in osteosarcoma
Source: BMC Syst Biol. 2012 May 28;6:50. doi: 10.1186/1752-0509-6-50 (PMC3431263; doi:10.1186/1752-0509-6-50)

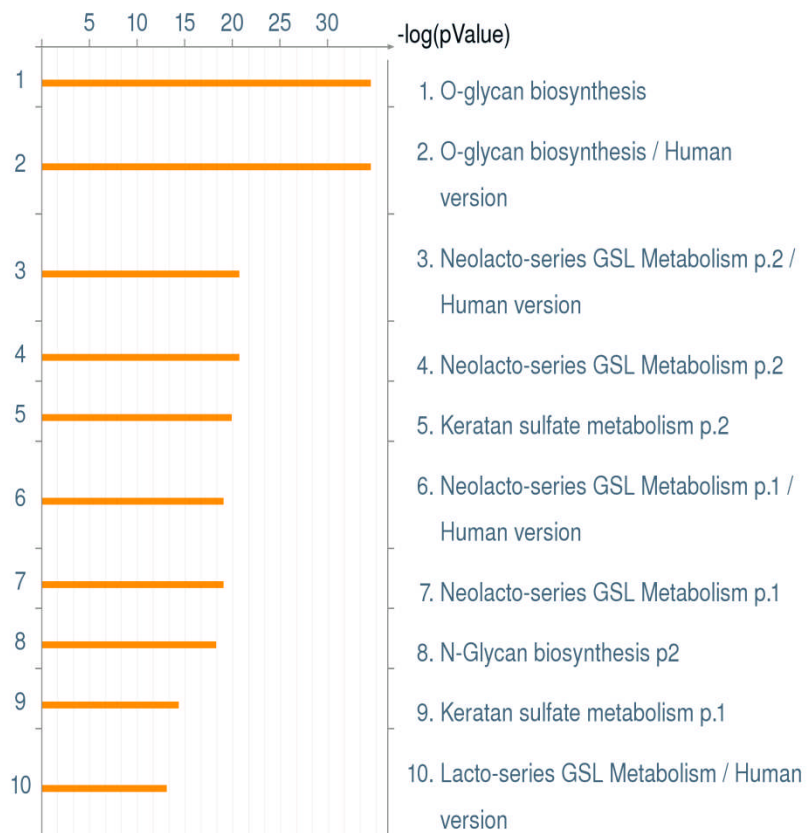

Supplement: Additional file 2 — Figure S1. Pathway analysis of all glycogenes. Top significant pathways identified by MetaCore using all 191 glycogenes identified in the genomic profile. All pathways shown are significant. Refer to Figure 1 legend for graph details. [file 1752-0509-6-50-S2.pdf]

## a. 143B vs. HOS

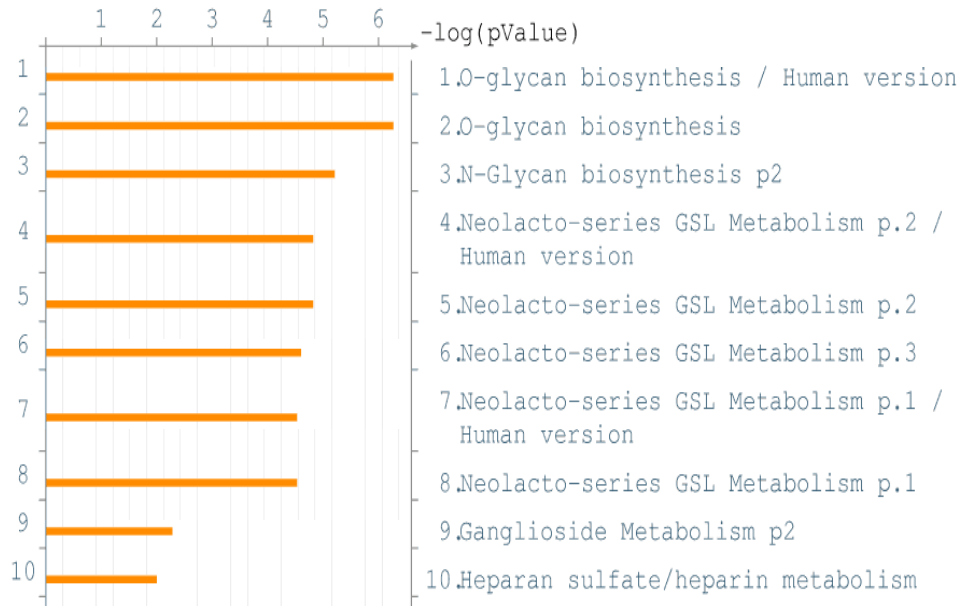

## b. LM7 vs. SaOS-2

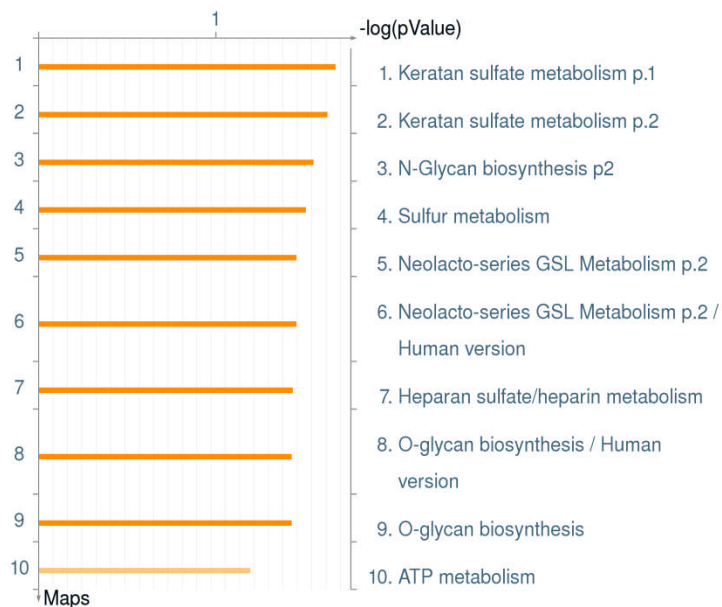

Supplement: Additional file 3 — Figure S2. Pathway analysis of differentially regulated glycogenes from 143B/HOS and LM7/SaOS-2 models. Top significant pathways identified by MetaCore using (a) differentially regulated genes from 143B/HOS model, and (b) differentially regulated genes from LM7/SaOS-2 model. Results showed that “N-Glycan biosynthesis” was the top common pathway between the two models. Dark orange bars represent significant pathways. Refer to Figure 1 legend for graph details. [file 1752-0509-6-50-S3.pdf]
