# Supplementary material for: A systems biology approach reveals common metastatic pathways in osteosarcoma
Source: BMC Syst Biol. 2012 May 28;6:50. doi: 10.1186/1752-0509-6-50 (PMC3431263; doi:10.1186/1752-0509-6-50)
Supplement: Additional file 4 — Methods for the N-Linked glycoproteins enrichment by lectin affinity chromatography. [file 1752-0509-6-50-S4.doc]

**Supplemental Results**

**N-Linked Glycoproteins Enrichment by Lectin Affinity Chromatography**

Lectins are proteins able to specifically and reversibly bind carbohydrates, and enable enrichment and characterization of different classes of glycoproteins such as mannose-rich glycan moieties. Different commercially available lectin-based kits were tested and the respective efficiency of these chromatography methods was calculated using the following formula: (total eluted protein) / (total protein loaded into the chromatography) x 100. After an initial testing, WGA lectin affinity column (Pierce, USA) with specificity for N-acetylglucosamine was selected for further characterization including testing the capturing efficiency of a known glycoprotein control. In order to test the capturing efficiency of a WGA specific glycoprotein vs. WGA non-specific glycoproteins, three different glycoproteins were used. First, bovine Fetuin was used as a WGA specific glycoprotein as it is a well-characterized glycoprotein with 3 N-linked and 4 O-linked glycosylation with a known high affinity to WGA lectin. Next, Transferrin and Lactoferrin where used as non WGA-specific glycoproteins. These two glycoproteins have high affinity for other types of lectins, but a lower affinity to WGA lectin relative to Fetuin. The lectin chromatography efficiency for pure samples of Lactoferrin and Transferrin was 3.9 and 3.7% respectively, while efficiency for pure bovine Fetuin was 33%. The WGA lectin chromatography efficiency was then tested on the human OS cell line lysate. To test the WGA lectin chromatography efficiency on the human OS cell line lysate proteins, a total of 1 to 1.5 mg of protein from each cell line was obtained and loaded into the lectin columns. The calculated WGA lectin efficiency using the total protein obtained from the OS cell lines was consistently 8 to 10%, with an adequate amount of captured glycoprotein for downstream analysis.
