# Supplementary material for: A systems biology approach reveals common metastatic pathways in osteosarcoma
Source: BMC Syst Biol. 2012 May 28;6:50. doi: 10.1186/1752-0509-6-50 (PMC3431263; doi:10.1186/1752-0509-6-50)

a. Pathway analysis of a randomly selected genes set

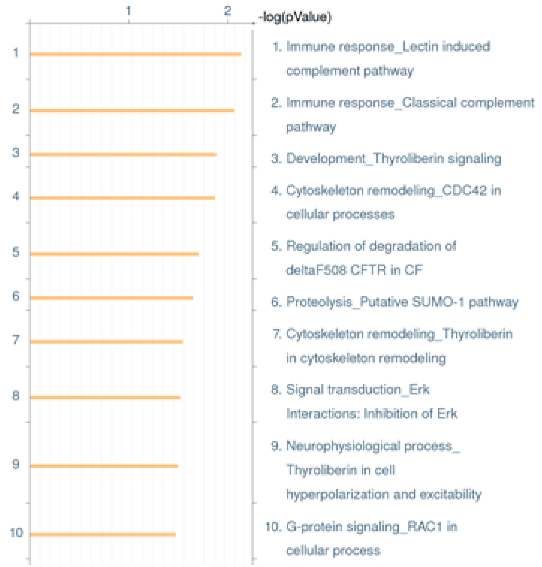

b. Pathway analysis of the Topological Nodes identified from the random genes set

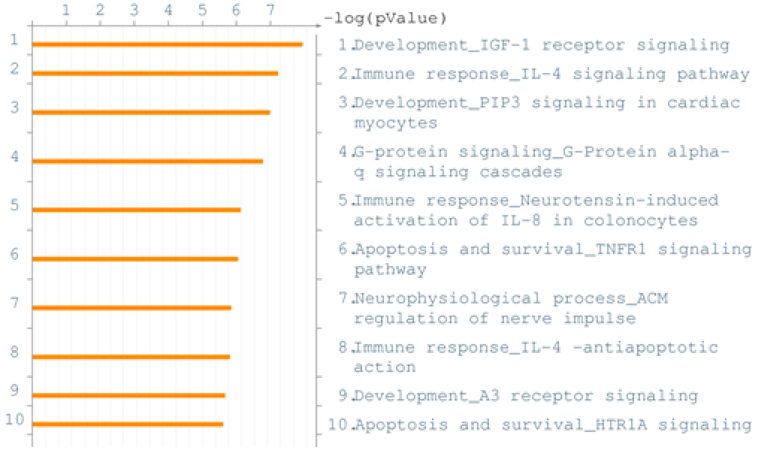

Supplement: Additional file 5 — Figure S3. Pathway analysis of random genes and their topological nodes. Top significant pathways identified by MetaCore using (a) 300 randomly selected genes and (b) topological significant nodes from the 300 randomly selected genes. None of the top common significant pathways identified from the topological analysis of the up-regulated genes and up-regulated glycoproteins from the 143B/HOS and LM7/SaOS-2 models were identified by the topological analysis of this random gene set. Dark orange bars represent significant pathways. Refer to Figure 1 legend for graph details. [file 1752-0509-6-50-S5.pdf]
